# Functional Characterization of Cotton GaMYB62L, a Novel R2R3 TF in Transgenic Arabidopsis

**DOI:** 10.1371/journal.pone.0170578

**Published:** 2017-01-26

**Authors:** Hamama Islam Butt, Zhaoen Yang, Eryong Chen, Ge Zhao, Qian Gong, Zuoren Yang, Xueyan Zhang, Fuguang Li

**Affiliations:** State Key Laboratory of Cotton Biology, Institute of Cotton Research of CAAS, Anyang, China; Texas Tech University, UNITED STATES

## Abstract

Drought stress can trigger the production of ABA in plants, in response to adverse conditions, which induces the transcript of stress-related marker genes. The R2R3 MYB TFs are implicated in regulation of various plants developmental, metabolic and multiple environmental stress responses. Here, a R2R3-MYB cloned gene, *GaMYB62L*, was transformed in Arabidopsis and was functionally characterized. The *GaMYB62L* protein contains two SANT domains with a conserved R2R3 imperfect repeats. The *GaMYB62L* cDNA is 1,017 bp with a CDS of 879, encodes a 292-residue polypeptide with MW of 38.78 kD and a pI value of 8.91. Overexpressed *GaMYB62L* transgenic Arabidopsis have increased proline and chlorophyll content, superior seed germination rate under salt and osmotic stress, less water loss rate with reduced stomatal apertures, high drought avoidance as compared to WT on water deprivation and also significant plant survival rates at low temperature. In addition, overexpressed *GaMYB62L* lines were more sensitive to ABA mediated germination and root elongation assay. Moreover, ABA induced *GaMYB62L* overexpression, enhanced the expression of ABA stress related marker genes like *RD22*, *COR15A*, *ADH1*, and *RD29A*. Together, overexpression of *GaMYB62L* suggested having developed better drought, salt and cold tolerance in transgenic Arabidopsis and thus presented it as a prospective candidate gene to achieve better abiotic stress tolerance in cotton crop.

## Introduction

Environmental abiotic factors especially drought stress severely effects the global production and distribution of important cash crops [1, 2]. To overcome these adverse conditions plants have evolutionarily developed a signaling cascade of relaying stress sensors, signaling transduction pathways, TFs and downstream targets, with an output of regulatory gene products and metabolites. Various intracellular molecular switches helps in adaption of plant to stress responses, by modulating transcript level of target genes which in turn brings physiological changes like up regulation of phytohormone, high levels of osmolytes, antioxidants and reduction in energy consumption [3, 4].

To comprehend the molecular mechanism of target genes in relation to drought tolerance, mining of new gene is imperative with anticipation of functional genomics and comparative genomics studies. TF works by the specific binding of the *cis-*acting element with the activated *trans-acting* element, in the target gene promoter region. To date, numerous stress responsive marker genes and promoter genes related to abiotic and biotic stress have been broadly researched in important agronomic plants [5, 6]. Based on DNA-binding domains (DBD) TFs are divided into MYB/MYC TFs, DREB TFs, WRKY TFs, NAC TFs, AP2/ERF TFs and bZIP TFs [7, 8].

MYB TFs are well known for significantly involving in drought responsive genes crosstalk or signaling pathways. It contains a conserved DBD at N-termini known as MYB domain with 53 a.a residues and a helix-loop-helix organization, and recently also discovered to be located at C-termini of MYB protein. MYB proteins are further divided into four types based on DBD repeats; MYB-1R, R2R3-MYB, R1R2R3-MYB (MYB3R), and 4-RMYB proteins [9, 10].

MYB TFs, vastly represented within dicots and monocots, accounts approximately 205 putative R2R3 MYB genes in cotton D genome which is highest number so far as compared to 126 in Arabidopsis [11, 12]. R2R3 MYBs are found engaging in numerous stress responses of plant development and intracellular metabolism processes [13, 14].

R2R3 MYB TF are accounted to implicate in diverse plant responses to abiotic stress condition like *AtMYC2/AtMYB2*, are well-known to participate important function in high ABA dependent response to water deficit and NaCl stress. The reported *AtMYB102* protein is an important component to amalgamate wounding, osmotic stress, and ABA transduction pathways in overexpressed transgenic Arabidopsis [7, 15]. Moreover *AtMYB60*, *AtMYB61*, *AtMYB44* and *AtMYB96* significantly regulate stomatal movements to decrease water loss during dehydration [16, 17]. *AtMYB41* is only involved in the short term transcriptional response whereas *AtMYB15* is recognized to confer osmotic tolerance in transgenic plants by up-regulated ABA levels [18, 19]. Similarly, *TaMYB33* [20], *NbPHAN* [21], *AtMYB20* [22], *MdSIMB1* [23], *OsMYB2* [24], *GmMYBJ1* [25], *GmMYBJ2* [26], *GbMYB5* [27] and *SbMYB8* proteins [28] are documented particularly to be positively implicated in transgenic plants responses to drought and multiple abiotic stresses.

Cotton is a key textile fiber and an important oil seed cash crop [29]. After the release of diploid cotton *Gossypium arboreum* genome sequence, it is documented to contains more than 200 R2R3 MYB genes [12, 30], thus it is imperative to research some novel genes which holds elite functionality against abiotic stress especially drought which is a serious threat to cotton production. Very few drought stress studies found in cotton, for instance, R2R3 MYB *GbMYB5* [27] than R2R3 cotton MYB proteins *GhMYB25*, *GhMYB25L* and *GhMYB109;*functions in early fiber and trichome development [31, 32]. Furthermore, the molecular co-ordinations and the plant gene behavior in relation to drought tolerance are still unclear.

Here, we transformed a novel *Gossypium arboreum* R2R3 MYB TF gene, *GaMYB62L*, in Arabidopsis and its ectopic overexpression results in enhanced tolerance against drought stress possibly due to high ABA phytohormone level, high rates of stomatal apertures closure with lower water loss rates, improved RWC, high proline levels and with good plant growth. Our results presented here provide a potential novel contender gene designated as *GaMYB62*L isolated from *G*.*arboreum* for future molecular modifications of drought-stress tolerant gene in cotton.

## Results

### *GaMYB62L* sequence characterization and phylogenetic analysis

**Fig 1:**
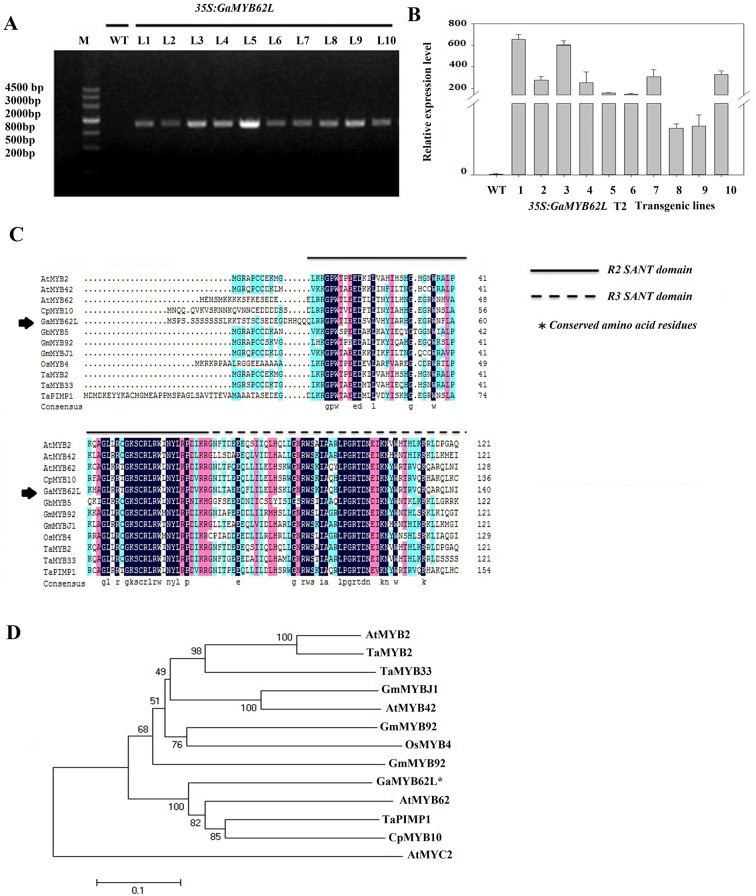
Multiple sequence alignment of *GaMYB62L* protein and Phylogenetic tree analysis. **(A)** PCR analysis performed to check 879 bp CDS integration in transformants from T_1_ generation. *35S* primer was used as a forward primer and reverse primer for respective gene was used for transformation confirmation. PCR products from DNA of *A*. *thaliana* Col-0 leave were used as the negative control (lane 1) and extracted leave DNA of *GaMYB62L* transgenic as test lines (lanes 2–11).M, kb DNA marker III. **(B)** Transcripts levels of the *GaMYB62L* of T_2_ transgenic lines tested by quantitative real-time PCR. *Arabidopsis Ubiquitin* 10 gene (*Accession no*: AT4G05320) used as internal standard. Three biological replicate data was represented with the mean ± SD value. **(C).** Amino acids alignment of *GaMYB62L* with other R2R3-MYBs plant: *GmMYBJ1* (AGO06072.1), *AtMYB42* (AEE83118.1), *GbMYB5* (AEF14025.1), *GmMYB92* (ABH02844.1), *TaMYB2* (AAT37168.1), *TaMYB33* (AEO21928.1), *OsMYB4* (BAA23340.1), *CpMYB10* (AAM43912.1), *AtMYB2* (BAA03534.1), AtMYB62 (AT1G68320) and *TaPIMP1* (ABU93236.1) **(D)** *GaMYB62L* proteins tree analysis was built by using MEGA 6.0 program with bootstrap values of (1000 replicates) which are indicated on the branches. The *AtMYC2* with gene bank accession number (EFH69991.1) was used as an out group in tree analysis. The predicted *GaMYB62L* was represented with (*Asterisk*). The accession numbers are indicated with deduced plant sources.

Our laboratory previous studies on RNA-seq data of *Gossypium arboreum* revealed to have hormone crosstalk, when MYB TF are implicated in modifying plant response toward drought and NaCl stresses in different tissues [33]. Thus based on these studies, novel cloned cotton gene *GaMYB62L* functional characterization was performed in Arabidopsis for drought stress. The full length CDS of *GaMYB62L* is 879 bp and contains a 292-amino acid harboring an expected MW of 38.78 kD with a theoretical isoelectric point of 8.91 (http://web.expasy.org.) (S1E Fig). Performing SMART analysis, (http://smart.embl-heidelberg.de/smart.), the deduced 292 residue polypeptide was determined to consist of two conserved SANT domains (MYB domains) as shown in (S1B Fig). The position of the putative TMH calculated by TMHMM 2.0 is shown in (S1D Fig). Hydropathy analysis revealed that *GaMYB62L* contains no TM domain, representing that *GaMYB62L* is not a TM protein. The secondary structure of *GaMYB62L* was analyzed by GOR ExPASy online tool, demonstrating that *GaMYB62L* protein includes 40.83% of α helix, 40.24% of random coil and 18.93% of extending chain (S1F Fig). The alignment results between cDNA and genomic sequence retrieved from cotton genome project database available online (http://cgp.genomics.org.cn/page/species/blast.jsp.) demonstrated that *GaMYB62L* contains 3 introns (S1C Fig) and is located on chromosome 6 with cotton ID number cotton_A_01409. The *GaMYB62L* protein alignment with other R2R3 MYBs plant: *GmMYBJ1* (AGO06072.1), *AtMYB42* (AEE83118.1), *GbMYB5* (AEF14025.1), *GmMYB92* (ABH02844.1), *TaMYB2* (AAT37168.1), *TaMYB33* (AEO21928.1), *OsMYB4* (BAA23340.1), *CpMYB10* (AAM43912.1), *AtMYB2* (BAA03534.1), *AtMYB62* (AT1G68320) and *TaPIMP1* (ABU93236.1) showed the presence of conserved R2 and R3 domains of MYB gene (http://www.ebi.ac.uk/Tools/msa/muscle/) (Fig 1C). Phylogenetic analyses indicated that *GaMYB62L* clusters with the R2R3-type MYB homolog of Arabidopsis (*AtMYB62*) which is previously known for having positive response to salicylic acid and also involved in phosphate starvation responses [34, 35], *TaPIMP1* which contributes host defense response to fungus *Bipolaris sorokiniana* and water deficit stress [36], and *CpMYB10* which meditates drought stress tolerance, modify ABA and glucose signal transduction response in transgenic mutants [37]. While R2R3 MYB genes of wheat (*TaMYB33* and *TaMYB2)* [20, 38] and *AtMYB2* [7] were presented together in a clade and known to involved in salt, drought and osmotic stress tolerance respectively. *AtMYB2* along with *AtMYC2* act as TF activators in ABA induced gene transcripts in plants on encountering drought stress, thus take part during osmotic stress responses. *GmMYB92* and *GmMYBJ1* from soybean were present in different clades and were known to involve in salt and drought tolerance respectively. Similarly, *AtMYB42* involved in cell wall production, *OsMYB4* participates in chilling and freezing tolerance and *GbMYB5* in drought stress tolerance respectively [39, 40, 41] (Fig 1D). In context to the tree analysis some of the homologue R2R3 MYB proteins (*TaMYB33*, *TaMYB2*, *TaPIMP1* and *GmMYBJ1*) were known to contain MBS *cis-*element in the promoter region which is involved in drought stress tolerance. Moreover, bioinformatic analysis of *GaMYB62L* promoter also predicted to contain MBS *cis*-elements [42], which might be involved in drought stress tolerance. Moreover, it also contains TC-rich repeats [43], HSE [44], WUN–motif (wound response element) [45], Box-W1 (fungal elicitor responsive element) [46] and *cis-*elements related to phytohormone like *MeJA* responsive *cis*-elements (TGACG-motif and CGTCA-motif) [47] as shown in (S1 Table).

### *GaMYB62L* overexpression positively modulates drought tolerance in transgenic lines of Arabidopsis

**Fig 2:**
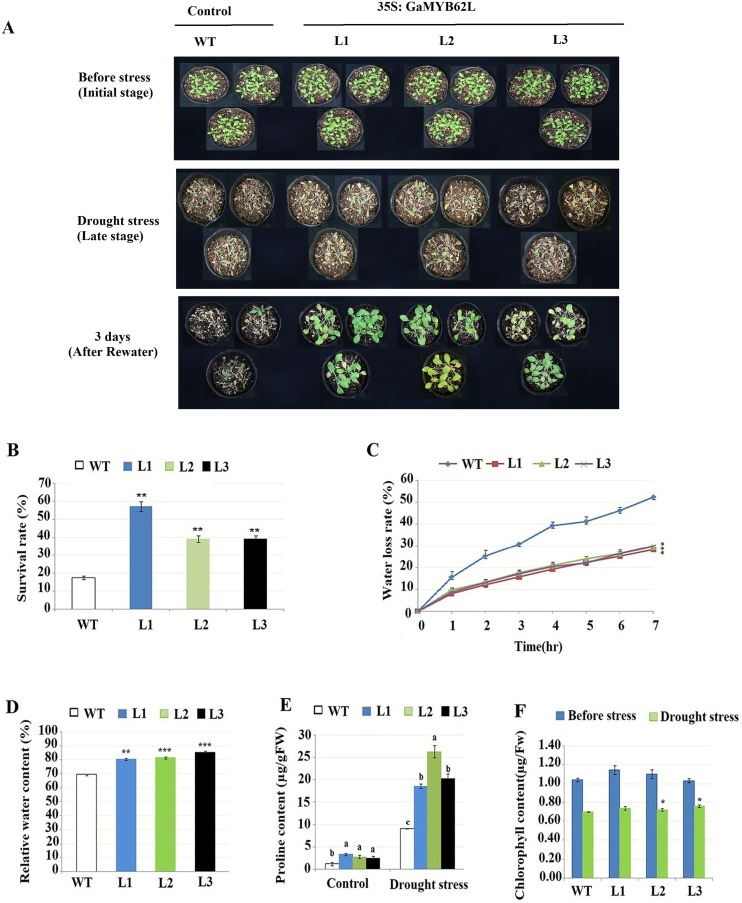
Water withhold assay, water status analysis and metabolite alternation of *35S*:*GaMYB62L* in Transgenic *A*. *thaliana* under drought stress. **(A)** Drought tolerance assay of *35S*:*GaMYB62L* overexpressed plants and WT. One week-old *35S*:*GaMYB62L* overexpressed plants, were transplanted to soil to grow for another 2-weeks (initial drought stress stage). Thereafter, transgenic plants were water withheld for 14 d (late stage of osmotic stress) for water deficit stress assessment. The *35S*:*GaMYB62L* overexpressed plants seedlings and WT were photographed at the initial and late stages of dehydration and rewatering. **(B)** The survival rate (%) was mean of three biological replicates (*n* = 18) with ± SE value.*p<0.05 and**p<0.01. **(C)** Leave water loss time course in detached leaves of 3 week old *35S*:*GaMYB62L* plants and WT, expressed as (%) of initial FW at designated time interval, significant value (*p <0.05) of three replicates means with ± SE (*n* = 10). **(D)** *35S*:*GaMYB62L* and WT relative water contents in 4 week old detached leaves from plants grown in pots, were left on a bench with RH 45–50% at 22°C and weighed. The leave was submerged in water to get turgid weight, afterwards dried at 80°C for 72 h for dry weight analysis. The Relative Water Content (%) = (TW—DW) / (FW—DW), mean of ± SD (n = 10 plants).* *p* <0.05, ** *p* <0.01, *** *p* <0.001, t-test. **(E)** *35S*:*GaMYB62L* lines free proline content was higher than WT plants in osmotic stress. Proline content was measured spectrophotometrically in 14 days of water deficit leave samples. The biological replicates mean value was calculated with± SE (*n* = 3). Duncan’s multiple range tests analysis labeled with similar letters are not significantly same. **(F)** The total chlorophyll content in transgenic plants and WT under drought stress. The total chlorophyll level was determined in 14 day drought stressed samples, (*n* = 3) ± SE with (*p <0.05) by t-test.

To dissect *GaMYB62L* possible function, transformed homozygous lines were generated in *A*. *thaliana* containing *CaMV-35S* promoter driven *GaMYB62L*. The T_1_ transformants were checked by PCR to check the positive integration of 879 bp CDS in the vector, for it *35S* primer was used as a forward primer and reverse primer of the respective gene (S3 Table), whereas *A*. *thaliana* (Col-0) leave DNA was used as a negative control (Fig 1A). T_2_ seeds were placed on selective medium BASTA agar plates and checked for ratio of surviving to death plants (S2 Table). According to Mendel's law of segregation, the lines with a segregation ratio of approximated 3:1 were probably having one insertion of T-DNA in the genomic, in which the expression was detected by qPCR (Fig 1B). The seeds of lines with relative higher gene expression level were obtained and successively selected on selective medium BASTA agar plates until we got homozygous lines. Three T_3_ generation lines (L1, L2 and L3) were selected for further analysis. To characterize the drought tolerance of transgenic *GaMYB62L* plants, 7-days *35S*:*GaMYB62L* and WT plants were relocated from MS medium to well watered soil and grown for further 2 weeks. Afterward, plants were withheld from water for 14 days to induce drought stress. After indicated time span pots were rewatered and results were recorded after 3 days (Fig 2A). Before rewatering, wild-type (WT) and transgenic plants showed apparent drought stress symptoms like drying and wilting. After rewatering 40–60% of *35S*:*GaMYB62L* plants survived in contrast to WT plants, with only 20% survival rate (Fig 2B). The water loss rate (WLR) of the WT and transgenic lines (L1, L2 and L3) were evaluated for over 7 hour (Fig 2C) Starting at 0.5 to 4 h, the WLR of the *35S*:*GaMYB62L* lines was considerably slower than WT, the water losses of *GaMYB62L* overexpressing lines leaves were 32, 30 and 28% respectively, whereas those of the WT leaves were 52%. The final relative water content (RWC) maintained by *35S*:*GaMYB62L* plants were 80–85% in contrast to 69% of WT, which is much higher in overexpressed plants as revealed in (Fig 2D). The above outcomes, indicates that *GaMYB62L* protein contributes drought tolerance in transgenic Arabidopsis probably by enhanced water retention ability.

### Analysis of proline and chlorophyll content in *GaMYB62L* transgenic lines

Free proline was well known to act as a multifunctional osmoprotectant, found ubiquitously and accumulates in plants under abiotic stress. Proline can participate significantly in attainment of osmotic stress tolerance [48]. The transgenic and control plants were given 14 days drought treatment, endogenous free proline content was drastically elevated in overexpressed *GaMYB62L* lines than that of WT (Fig 2E), this suggests that *GaMYB62L* overexpression led to enhanced endogenous proline synthesis which results in better drought tolerant transgenic Arabidopsis plants.

Subsequently, under normal condition *35S*:*GaMYB62L* lines and WT showed no clear significant differences in chlorophyll content, but in 14 days drought stressed samples, overexpressed L1, L2 and L3 had significantly higher content of chlorophyll as shown in (Fig 2F). So, these significant levels in overexpressed transgenic plants strengthen the speculation that because of less damage to photosynthetic apparatus, transgenic plants maintained stay green property, thus distinguished its role in drought stress tolerance.

### Constitutive expression of *35S*:*GaMYB62L* in arabidopsis up-regulates tolerance to multiple abiotic stresses

**Fig 3:**
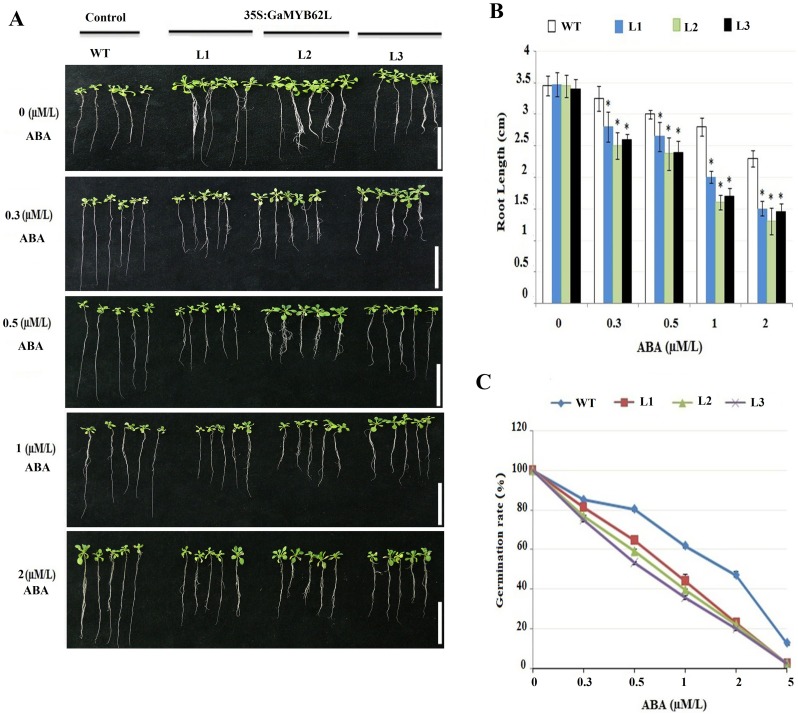
Increased sensitivity to ABA inhibition of root elongation in *GaMYB62L* overexpression seedlings and Germination rate. **(A)** Comparisons of root elongation between WT controls and *GaMYB62L* overexpression lines seedlings on MS with and without different ABA conc. (μM/L). Four day old seedlings were shifted to 0.5 MS plates containing varied ABA conc. and grown for 7 days. Line bar: 1 cm. **(B)** Quantitative comparisons of the differences of root elongation between WT controls and *GaMYB62L* overexpression lines seedlings on the MS having 0, 0.3, 0.5, 1 and 2 ABA conc. (μM/L), respectively. The values were calculated using the data from three separate root growth assays (with at least 30 seedlings examined per genotype per ABA concentration per assay). Samples mean with ± SE (*n* = 3) were calculated by t-test (*p <0.05). **(C)** *35S*:*GaMYB62L* plants hypersensitivity to varied ABA concentration. *GaMYB62L* overexpressed and WT plants germination rates on MS with and without varied ABA conc. (μM/L), the results were scored at 10 days. 50 from each transgenic line and WT were used, ± SE (*n* = 3).

Next, we examined whether *GaMYB62L* was implicated in ABA, salt, and osmotic stresses. The seeds from both transgenic and control were grown on MS media with different ABA concentration (0μM, 0.3μM, 0.5μM, 1μM, 2μM), NaCl (0, 50mM, 100mM, 125mM and 150mM) and Mannitol (0 mM, 100 mM and 200 mM) and grown for 10 days. The *35S*:*GaMYB62L* lines were hypersensitive to ABA induced inhibition than control plants. Subsequently, 2μM ABA revealed decreased seeds germination rate for transgenic plants nearly upto 20%, while WT seeds retained 50% germination rate under the similar conditions (Fig 3C). Also in root length assay, varied ABA concentration revealed significant sensitivity of root length of transgenic *GaMYB62L* seedlings as compared to WT (Fig 3A and 3B), which depict that ABA is involved in root development process and also strengthens the speculation that this gene follows and might be regulated in ABA dependent manner.

**Fig 4:**
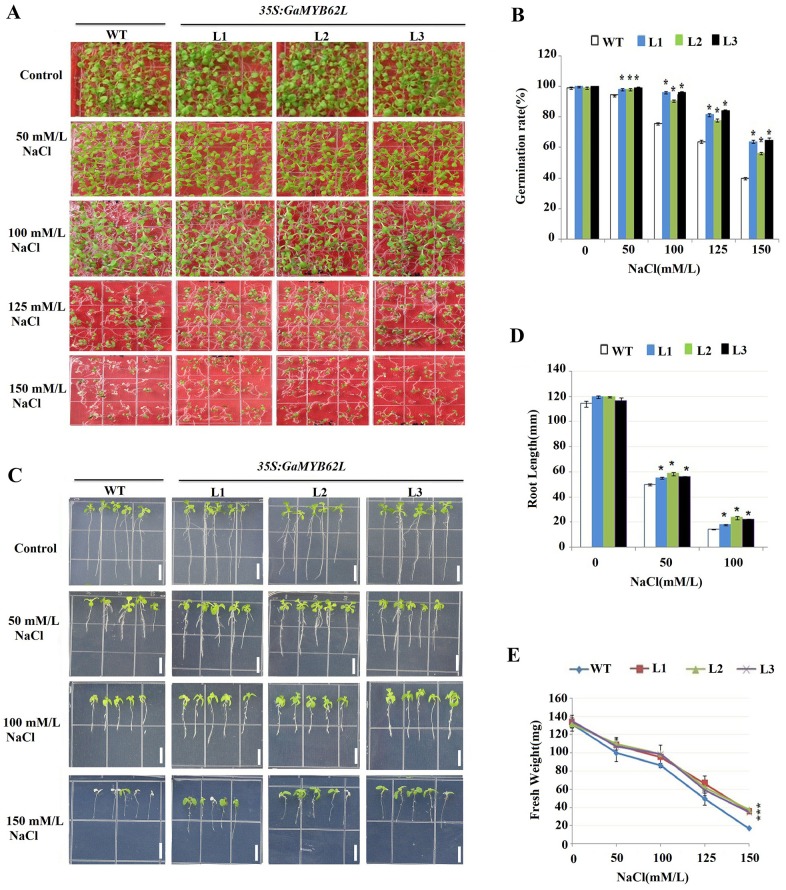
Seed germination assays of *GaMYB62L* overexpressed lines and WT in response to NaCl. **(A)** *35S*:*GaMYB62L* lines (L1, L2 and L3) and WT seedlings growth performance germinated on MS media in presence and absence of 0, 50, 100, 125 mM and 150 mM NaCl. **(B)** The seed germination ratio of *35S*:*GaMYB62L* overexpressed plants and WT on MS NaCl (0, 50, 100, 125 and 150). Seed germination rate was monitored with full radicals penetrating from the seed coat, at 10^th^ day of post stratification. The germinated seeds with albino phenotype were considered dead. 50 seeds from each transgenic and control plants were used with biological replicates (*n* = 3) and means of ± SE, with (*p <0.05, t-test). **(C)** Comparisons of root elongation between seedlings of WT and *GaMYB62L* overexpression lines on the medium without and with different ABA conc. (μM/L). Four day old seedlings were placed on 0.5 MS plates supplemented without and with varied NaCl conc. for 6 days. Line bar: 1 cm. **(D)** Quantitative comparisons of the differences of root elongation between WT control and *GaMYB62L* overexpression lines seedlings on MS media without and with 0, 50, 100, 125 and 150 mM NaCl, respectively. The values shown were each calculated using the data from three separate growth assays (with at least 30 seedlings examined per genotype per NaCl concentration per assay). (*p <0.05, t-test), with biological replicates ± SE (*n* = 3). **(E)** Fresh weight of WT and *GaMYB62L* overexpression lines seedlings germinated on MS media without and with 0, 50, 100, 125 and 150 mM NaCl. The FW was determined at 10^th^ day of germination. Data represented are means ± SE of 3 replicates, (10 seedlings) each.

**Fig 5:**
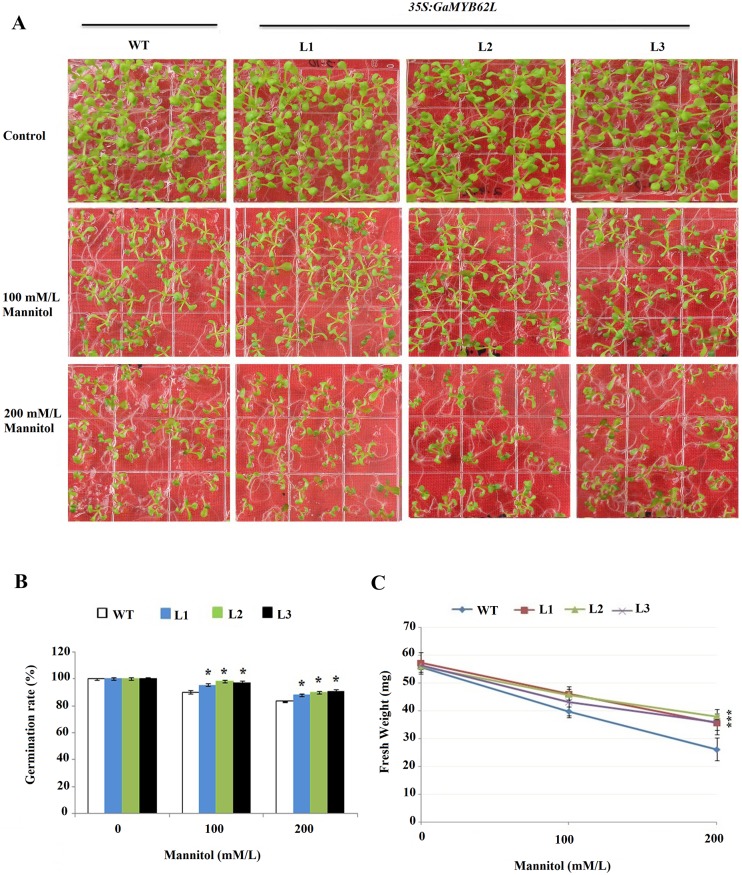
*35S*:*GaMYB62L* overexpressed lines and WT Seed germination assays in response to osmotic stress. **(A)** *35S*:*GaMYB62L* overexpressed lines seedlings and WT growth performance germinated on MS media with and without 100 or 200 mM mannitol. After 10 days of germination plates were photographed. **(B)** Seed germination ratio of *35S*:*GaMYB62L* overexpressed lines and WT on MS media in the absence and presence of 100mM mannitol or 200mM mannitol. Data are the means of ±SE of 3 replicates, with 36 seeds of each plant (*p <0.05). **(C)** Fresh weight of *GaMYB62L* overexpression lines and WT seedlings, germinated on MS media with and without 100 or 200mM mannitol. The FWs were measured at 10^th^ day of germination. The data means (±SE) of 3 replicates with (10 seedlings) each.

In 150mM NaCl seed germination rates of *35S*:*GaMYB62L* lines were higher with greater fresh weight as compared to WT, which underwent delayed growth rates with subsequent pale colored cotyledons and some of them unable to germinate (Fig 4A, 4B and 4E). Normal culture conditions did not have any obvious contrast during seed germination of the transgenic L1, L2 and L3 as compared with WT. Moreover, transgenic lines (L1, L2 and L3) maintained healthy root growth in 100mM NaCl plates (Fig 4C and 4D). When 200mM Mannitol was used, the growth rate of transgenic seedlings were higher and had normal shaped rosettes than WT (Fig 5A and 5B), also in comparison to WT transgenic plants had more fresh weight (Fig 5C). Overall, the growth of *35S*: *GaMYB62L* plants were good in comparison to WT plants that showed slight inhibition, suggesting the involvement of transgene in a general osmotic response that make it more drought-tolerant.

### Reduced leaf stomatal density, size and stomatal opening in *35S*: *GaMYB62L*

**Fig 6:**
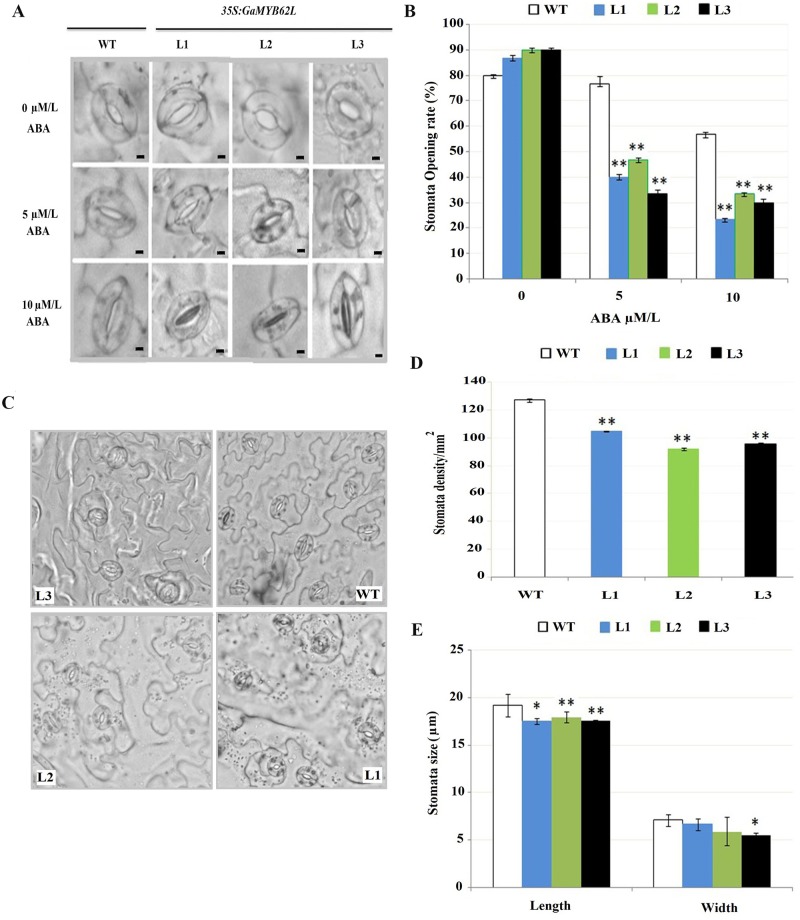
Stomatal changes in WT and *35S*:*GaMYB62L* overexpressed lines in response to ABA. **(A)** Quantitative comparisons of stomata aperture differences of WT and *35S*:*GaMYB62L* overexpressed lines (L1, L2, and L3). WT and *35S*:*GaMYB62L* lines photographed under control and two concentrations of ABA (5 and 10 μmol/L). The two genotypes stomatal aperture did not differ in the absence of ABA, but in presence of varied ABA conc. stomatal aperture reduction in both genotypes, with the degree of the reduction being substantially higher for *35S*:*GaMYB62L* lines than WT. The reduction of stomatal aperture by ABA was expressed as percentage of control, which was taken as 100% for both genotypes. The values shown are the means of three 3 replicates (*n* = 10). Bars, approximately 1μm. **(B)** Stomatal opening rate comparisons of *GaMYB62L* overexpressed plants and WT, when treated with varied ABA concentrations. The stomatal apertures opening rates were calculated after 2.5 h of treatment with different ABA conc. 3 plants with 5 different sites were examined and photographed, guard cell apertures were digitally measured (ImageJ software). Aperture sizes lesser then 0.1 μm were measured as closed, significant difference value were calculated by (*p < 0.05, ** *p* <0.01). **(C)** Leave stomatal morphological phenotype and density of *35S*:*GaMYB62L* and WT plants photographed under OLYMPUS Bx51 microscope with 40x magnification. **(D)** Comparisons of leave stomata density in *35S*:*GaMYB62L* overexpressed plants and WT. 5 sights and 3 plants replicates were observed microscopically for each of transgenic and WT plant, values were means of ± SE. Stomatal density analysis and guard cell aperture was done under OLYMPUS Bx51 microscope with 40x magnifications. **(E)** *35S*:*GaMYB62L* stomata size comparisons with WT (10 stomata for each of 5 microscopic sight). The 3 plants leaves for each line were monitored (*p < 0.05; ** *p* <0.01).

To explore if the stomatal aperture closure is sensitive to ABA, we examined the constitutive *GaMYB62L* overexpression on guard cell apertures in the leaves under a microscope (Fig 6A). Stomatal density of *35S*: *GaMYB62L* plants were 105, 92 and 96 per mm^2^ respectively as compared to WT, which was higher about 127 per mm^2^. The average stomatal length to width dimension was 19.2 by 7.12 μm for the WT, in contrast with 17.99–17.56 by 6.69–5.93 μm for transgenic lines as shown in (Fig 6C, 6D and 6E). ABA induction results in significant percentage of stomatal cell aperture reduction in overexpressed *GaMYB62L* plants, while on 5μM ABA treatment WT plant stomatal apertures opening percentage was 23.34% than *35S*:*GaMYB62L* plants, which was 53.4,66.67%, and 40–33% respectively (Fig 6B). The percentage of *35S*:*GaMYB62L* plants stomatal opening is further reduced to 23, 30 and 33% for transgenic L1, L2 and L3 as compared to 56.6% when treated with 10μM ABA. Thus, reduced guard cells and apertures of *35S*:*GaMYB62L* plants along with reduced size and stomatal density per mm^2^ displayed swift change in stomatal aperture reduction than WT plants; mainly due to abscisic acid response which therefore helps to decrease WLR with subsequent good drought tolerance in *GaMYB62L* overexpressed Arabidopsis plants.

### Cold response of *GaMYB62L* overexpressing Arabidopsis plants

**Fig 7:**
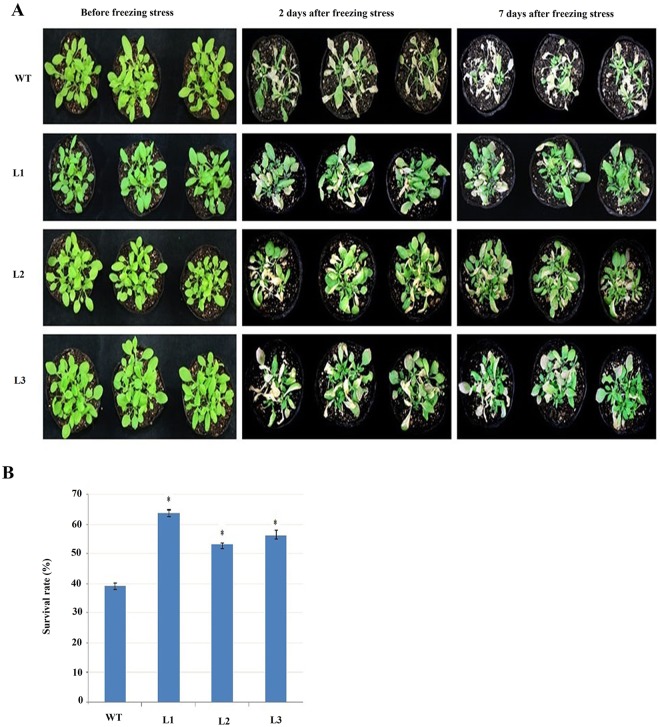
Responses of *GaMYB62L* overexpressed lines and WT plants to cold stress. **(A)** 3 week *GaMYB62L* overexpressed and WT plants were placed at –10°C for three hour, and then shifted to growth room for plant recovery. *35S*:*GaMYB62L* and WT plants were photographed after seven days. **(B)** The percentage of plant survival was determined as total number of phenotypically green revived plants. Experiment was run in triplicate with mean values ± SD (*n* = 18) and are significant (*p <0.05), t-test.

Overexpressed *OsMYB4* and *OsMYB3R-2* genes of rice were reported to be tolerant to low temperature mainly due to up-regulation of *COR* genes and high levels of osmoprotectants proline levels [41,49]. It revealed that high levels of compatible solutes like proline, underlies an important means which confers enhanced cold tolerance in rice MYB genes. The three week old *GaMYB62L* transgenic plants and WT plants were exposed for 3hr at –10°C and before shifted to growth room (22°C), kept at 4°C for 4 hour. After 2 days of revival under normal growth conditions 22°C±1, majority of *35S*:*GaMYB62L* lines seedlings were still green, while most of the WT plants turned pale yellow (Fig 7A). About 63.63,53 and 56% of transgenic plants (L1, L2 and L3) were able to survive respectively and grew as usual on recovery, while only 39% of the WT plants were able to survive (Fig 7B). The data imply that *35S*:*GaMYB62L* plants have augmented tolerance towards freezing stress in contrast to WT.

### Transcripts investigation of abiotic stress-responsive genes

**Fig 8:**
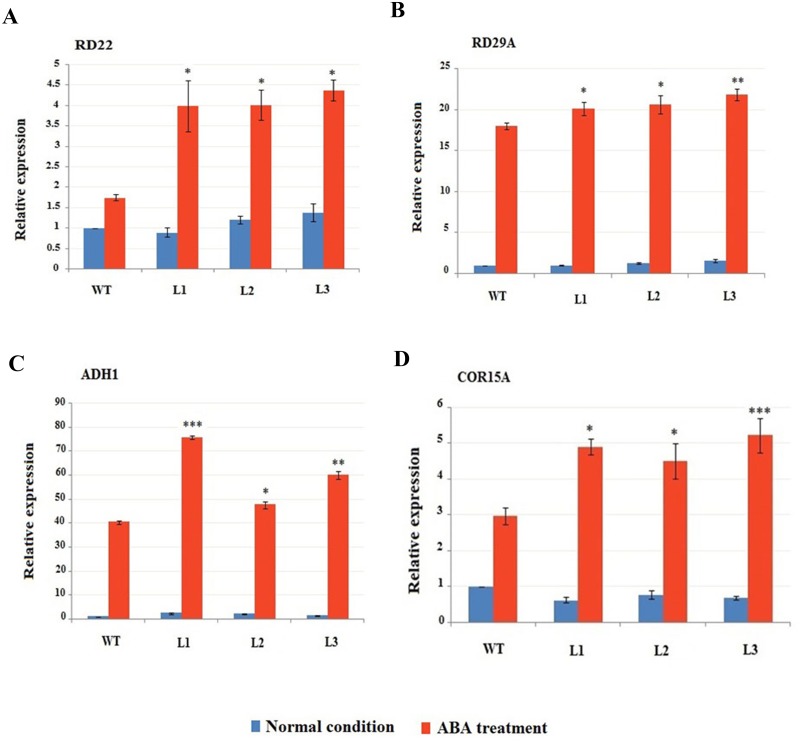
Transcript levels of stress-responsive genes in *35S*: *GaMYB62L* in response to ABA. **(A)** Transcript level of stress-responsive markers in *GaMYB62L* overexpressed and WT treated by 100μM ABA using qRT-PCR. Test and control samples (2 week old) were treated with and without 100μM ABA for 6 hr. Transcript levels of *RD22*, *ABI1*, *ABI2*, *ADH*, *COR15A*, *RD29A*, *RD29B*, *EM6*, *RD26* and *P5CS* stress marker genes were analyzed by qRT-PCR. Arabidopsis *ubiquitin 10* used as control gene, means value with ±SD. * *p* < 0.05; ** *p* <0.01 and *** *p* <0.001 calculated by student t-test.

Several prior studies have recommended numerous good stress related markers genes. Thus, in this study, we chose *RD22*, *ABI1*, *ABI2*, *ADH*, *COR15A*, *RD29A*, *RD29B*, *EM6*, *RD26* and *P5CS* as stress-responsive marker genes, and checked their transcripts in *GaMYB62L* and WT transgenic Arabidopsis, treated with and without 100μM ABA for 6 hr, in order to dissect the possible regulatory relationships of marker genes with *GaMYB62L*. Our qRT-PCR results showed that out of these genes *RD22*, *ADH*, *RD29A* and *COR15-A* responded well to ABA treatment than in WT plants (Fig 8A, 8B, 8C and 8D). Expression investigation of these up-regulated stress-responsive markers upon ABA proposed to have a close relationship with *GaMYB62L* protein in plant response towards abiotic stresses.

## Discussion

MYB transcription factor superfamily is large, with R2R3 MYB highly represented in higher plant species, also have a good role in gene expression regulation under different environment scenario, and display multiple roles in plants [14]. More recently, R2R3 MYB genes role in rice, wheat and soya have been demonstrated in multiple stress responses [20, 24, 25]. We study a new candidate gene of *Gossypium arboreum* from MYB TF family designated as *GaMYB62L*; it was transformed to get homozygous transgenic lines which were then validated for drought, salt and cold stress function. The amino acid sequence analysis and phylogenetic tree showed that *GaMYB62L* contains two highly conserved SANT-DNA binding MYB domains which grouped with R2R3-MYB from other plant (Fig 1C and 1D), indicating that *GaMYB62L* is a functional protein with an R2R3-MYB domain. The MYB have conservered *SANT* type domain carrying two repeat (R2,R3) which forms the DNA binding domain and identified in numerous chromatin regulatory proteins like *Swi3*,*Ada2*,*TFIIIB*,*Ncor* and *ISWI* proteins [50]. The *SANT-DBD* was also known to redirect MYB function in chromatin organization by histone modifications [51].

ABA is well-known to engage in numerous environmental stimuli related signaling pathways of plant species [52, 53]. The plants subjected under water deficit conditions, can produce and build up more abscisic acid with drought tolerant phenotype which is assisted by numerous stress tolerant biomolecules triggered by an individual TF. The drought related phenotypes comprises better and broad roots and compact guard cell density, which contributes to keep water balance and retain intracellular homeostasis of plants [54, 55], elevated proline content positively contributes to improved ROS detoxification and osmotic regulation [56, 57], moreover accumulation of ABA hormone help in decrease water loss rate from stomatal cell thereby make transgenic plants stress tolerant. The ectopic expression of transgenic plants also unravels enhanced response to stress responsive genes with increase transcripts levels as reflected by improved ABA contents and endogenous proline built-up [58].

To assess the role of *GaMYB62L* tolerance for numerous abiotic stress conditions, we make overexpressed transformed plants to understand the possible underlying molecular and physiological function of *GaMYB62L*. *35S*:*GaMYB62L* transgenic Arabidopsis as shown in (Fig 2A) have normal plant growth, which is in contrast to previous studies of *AtMYB41* which experienced stunted growth in overexpressed plants [59].

The seed germination rates are generally dependent on the water movements into the seeds, and to avoid low water potential upon abiotic stress, plant tissue keep a balance by turgor regulation to ensure continued plant growth and survival [60]. Thus, salt inductions at germination stage could results in both osmotic stress and specific ion toxicity [61, 62]. As *35S*:*GaMYB62L* transgenic plants showed higher rates of seed germination as compared to the WT under 200mM mannitol treatment, thus it depicts that the salt hypersensitive phenotype in transgenic plants is mainly because of the osmotic stress. These results corroborated with the function of *AtMYB102*, *AtMYB41* and *GmMYBJ2* which have been demonstrated to affect dehydration after wounding and implicated in ABA dependent osmotic and NaCl stress [15, 19, 26]. The high NaCl ionic toxicity could results in replacements of potassium ions with sodium ions inside the cell that brings conformational changes and interfered to regular biochemical pathways [63]. Therefore, *35S*:*GaMYB62L* transgenic plants might cope with this situation by adapting osmotic stress tolerance, Na^+^ ion extrusion and tissue tolerance. These adaptations mainly occur via osmotic adjustments with the exclusion of absorbed ions in vacuoles and accumulation of osmoprotectants in cytoplasm [64, 65]. Moreover, root growth is an important factor to endure salt stress as it is in direct contact with soil and provides important clue to response of transgenic plants to NaCl stress. As roots are more sensitive to Na^+^ ion stress thus *35S*:*GaMYB62L* transgenic plants shows significant root length growth upon NaCl treatment as compared to WT possibly due to germination differences with an increase in FW, which implies that ionic stress was manifested.

Exogenous biotic and abiotic stimuli results in built up of endogenous phytohormone ABA, which simulated as drought stress in the transgenic plants [3, 66]. On exposure to osmotic stress, transgenic plants showed varied degree of ABA sensitivity during different germination periods [67, 68]. Constitutive expression of *GaMYB62L* results in hypersensitivity to varied ABA concentration both in germination and root length assays than WT which are in line to the prior researches [18, 22, 69]. The consideration is that ABA-dependent pathway might be the major route for *GaMYB62L*, with enhanced tolerance against multiple stress condition, also there is a possibility that ABA might control the growth of roots by up regulated expression of *GaMYB62L*. Because of the ABA sensitive phenotypes at germination and post germination stages we further check the stomatal movements upon different ABA treatments. As elevated levels of abscisic acid underwent modifications for a set of physiological processes to handle unfavorable stressed conditions, thus we inferred that the *GaMYB62L* transgenic plants may have superior water retention abilities [70] under drought stress which led to descent in reduced transpiration rate, which contributes to reduced stomata density and reduced stomata apertures [71]. The *35S*:*GaMYB62L* plants have reduced stomatal size, stomatal densities and reduced rate of stomata opening than WT (Fig 6B, 6C and 6D), which corroborated the previous studies in *GbMYB5* where only stomatal densities are similar in transgenic and WT tobacco plants [27].

WLR is a key physiological indicator to elucidate the probable mechanisms of *GaMYB62L* protein that are implicated in improved osmotic tolerance of transformed plants [72]. In current study, the WLR was lower in leaves of transgene than WT ones (Fig 2C), which is in line to the recent studies of R2R3 MYB in wheat, cotton and soybean [26, 27, 73]. Also, *GaMYB62L* transgenic Arabidopsis have higher Relative Water Content % than control plants when prone to osmotic stress.

As *GaMYB62L* results in significant reduction of stomatal aperture upon ABA treatment which helps to reduce WLR in transgenic plants, so we further performed drought tolerance test. Drought stress can induce numerous phenotypical and physiological responses in plants as reported by [1]. After 14 days of water withholding, *35S*: *GaMYB62L* plants exhibited high survival rates than WT, which inferred that *GaMYB62L* protein is implicated in plant responses to water scarcity and can be utilized for future plant breeding improvements. These results corroborate previous studied roles of *AtMYB2*, *CpMYB10* and *TaPIM1* which clusters with *GaMYB62L* and reported to conferred multiple stress plant tolerance [7, 36, 37].

The high level of chlorophyll at all stages of plant life cycle is significantly associated with good transpiration efficiency, improved rates of photosynthesis and enhanced drought avoidance mechanism in water-deficit environment [74, 75]. To identify and assess the ability of transgenic plants ameliorating drought effects on photosynthesis, total chlorophyll level were determined after 14 days of water withheld. The loss of chlorophyll in overexpressed lines was significantly lower than WT (Fig 2F). Thus, these data suggest that photosynthetic machinery is protected from deleterious drought effects, possibly due to increase production of osmoprotectants proline. Accumulation of compatible solutes such as free proline is significant to sustain plant cell homeostasis and structural stability under water deficit, salinity and low temperature stress. Proline is an osmoprotective molecule which acts as a molecular chaperone, helps in decreasing cell water potential by increasing antioxidant function which protects the plant from harmful effect of multiple stresses [24, 37, 76, 77]. Proline content was significantly increased in *GaMYB62L* overexpressed lines following 14 days of drought stress than in WT (Fig 2E), suggesting that *GaMYB62L* can also regulates the proline synthesis in transgenic plant. The high proline results in context with good drought tolerance together recommended that *GaMYB62L* plants might have synthesize osmolytes to alleviate cell damage, this is useful to select this gene as a novel gene for cotton breeding.

TFs R2R3-MYB is probable to regulate a set of targeted marker genes when transgenic plants were induced by ABA. Thus, it was imperative to understand and determine the genes that interplay the stress tolerance phenotype in *35S*:*GaMYB62L* transgenic Arabidopsis. In an attempt to dig more about the downstream genes interactions which were regulated by *35S*:*GaMYB62L*, we investigated the expression pattern of 10 genes specifically for stress response upon ABA treatment. The *35S*:*GaMYB62L* over expression led to up regulation of 4 genes transcripts, *RD29A*, *RD22*, *ADH* and *COR15A* as compared to WT upon ABA treatment. Hydrophilic proteins *RD29A* and *COR15A* are known to exist in DRE or related motifs promoter regions which are specifically induced by multiple abiotic stresses [78, 79].

The *RD29A* can follow either ways of ABA-dependent or ABA-independent, when get activated by the combined binding action of DRE and ABRE *cis* motif in the gene promoter region [80]. Overexpression of DREB1A/CBF1 TF in *A*. *thaliana* led to up-regulation of numerous hydrophilic protein genes like *RD29A* and *COR15A* [81, 82]. Similarly, *TaMYB19* works in either manner of ABA transductions and aid in improved osmotic stress resistance specifically due to the amplified transcripts of *RD22* and *RD29A* [73]. The ABA-responsive expression of *RD22* is due to co-expression activation of both *AtMYB2* and *AtMYC2* in contrast to *ADH1* which only needs activated *AtMYB2* [7, 83]. Investigations of *AtADH1* promoter also depict the presence of (CACGTG, a MYC recognition site) G-box which is critical for gene initiation by ABA phytohormone [84] also reported for specific binding of *AtMYB2* to MYB recognition site in *AtADH1* promoter region, could trans activates gene upregulation by *MBS-1* site in transient transcripts monitoring. *AtMYC2* is well-known to binds specifically to the CACATG *cis* motif sequence that behave as a water deficit *cis*-element in the promoter regions [7]. Based on the above functional validation interpretations we speculate that *35S*:*GaMYB62L* plants holds good tolerance for drought, salt and cold stresses and upon ABA treatment it functions in ABA-dependent manner, moreover the enhanced osmotic stress tolerance of the *35S*:*GaMYB62L* transgenic plants are probably due to enhanced transcripts of *RD22*, *ADH1*, *RD29A* and *COR 15A*. Interestingly, bioinformatic analysis of *GaMYB62L* predicted to contain MBS, W-BOX and WUN cis-elements, P-BOX and CGTCA motif (for MeJA responsiveness), which warrants further investigation to understand the roles of these *cis-elements* in the transactivation and induction of *GaMYB62L* by ABA, biotic and abiotic stresses.

## Conclusion

Based on molecular and physiological analysis, we speculate that the high tolerance of *GaMYB62L* transgenic Arabidopsis, a novel R2R3 MYB to abiotic stresses might be because of shared role of up-regulated transcripts of downstream stress-responsive gene, high osmo-protectants proline levels, decrease water loss rate under ABA induced reduced stomatal apertures and more notable significant levels of chlorophyll which helps in reduced harm to photosynthetic machinery on exposure to abiotic stresses. In the support of above results, we recommend that the overexpression of *GaMYB62L* holds improved drought, salt and cold stress tolerance. Thus, these results have greatly enhanced our perception about possible function of *Gossypium arboreum* R2R3 MYB transcription factors in augmented plant response to environmental abiotic stresses and provide a novel contender gene for future cotton breeding advancement.

## Materials and Methods

### Plant transformation and screening of *35S*:*GaMYB62L* Arabidopsis lines

The *CaMV-35S* driven *pCAMBIA3300-GaMYB62L* construct in *Agrobacterium tumefaciens GV3101*, was confirmed by forward primer pair of *GaMYB62L* (TCTAGAATGAGATTAATCGGTGAGAT) and reverse primer pair of *GaMYB62L* (GAGCTCTCAATGCAATGCATTTCTAA). The *A*. *thaliana* ecotype (Col-0) wild-type plants (WT) were transformed by [85], floral dip method with minute modifications. Infiltration media used for transformation contains MS 2.215g/L, sucrose 50 g/L, MES 0.5/L, Silwet-77/L, 6-BA 0.01mg/L with pH 5.7. The transformed T_0_ Arabidopsis seeds were selected by directly growing seeds in the pots, followed by stratification for 2–3 days at 4°C to break dormancy and then later shifted in Arabidopsis growth room with 16hr light/8hr dark regime. The 7 days old seedlings were sprayed with 1% BASTA solution as shown in (S2A and S2B Fig). After second BASTA spray, 3–5 days later the survived positive seedlings were shifted to new pots and allowed to set seeds. The T_1_ transformed lines were confirmed by PCR Mighty Amp Genotyping Kit (Takara, China) using sense *35S* primer and antisense gene primer (S2C Fig).

The T_3_ homozygous progeny was bred from a T_2_ population, after qRT-PCR and selection of segregated lines with correct ratio (Fig 1B and S2 Table) using *qGaMYB62L* forward primer (GCCTGGAAGAACCGATAATGAGA) and *qGaMYB62L* reverse primer (GAGCTCTCAATGCAATGCATTTCTAA), with total cDNA as template. The phenotypic investigations were carried out on T_3_ or T_4_ homozygous lines.

### Response of overexpressed *GaMYB62L* transgenic lines to drought and cold stress

T_3_ homozygous transgenic lines were sterilized by 10% Bleach solution *(v/v)* for 10 minutes, and stratified for 3 days at 4°C before plating on MS. After 7 days seedlings were shifted to small pots, containing well watered equal amounts of (vermiculite: humus). After 2 week growth, pots were subsequently dehydrated for the next 2 weeks, afterwards pots were rewatered simultaneously and the plants revival rates were scored on day 3. The dehydrated plants were scored as survivals with green colored leaves, and the survival ratio were calculated and experiment was repeated thrice [86].

For freezing tolerance experiment three week transgenic and control WT plants were treated with −10°C for 3 hour and before shifted to Arabidopsis room, kept at 4°C refrigerator for 4 hour [69]. The survival rates were calculated based on greening phenotype, and healthy growing plants were counted as survival as compared to pale dead ones.

### Germination and root elongation assays

Germination assay was carried out by using seeds from WT and *GaMYB62L* transgenic lines that were sown on 0.5 MS, supplemented by varied Mannitol concentrations (0, 100mM and 200mM), NaCl (0, 50mM, 100mM, 125mM 150mM) [87] and exogenous ABA hormone (0, 0.3μM, 0.5μM, 1μM, 2μM) [18]. The plates were stratified for 3 days at 4°C before being shifted to 22°C. The plants phenotypes were examined at 10^th^ days of germination.

Transgenic lines (L1, L2 and L3) were checked for NaCl stress tolerance by first growing seedlings in vertical position on MS for at least 4 days and then shifted to the new plates with varied NaCl concentrations (0mM, 50mM, 100mM and 150mM) and ABA (0μm, 0.3μM, 0.5 μM, 1μM, 2μM), and continued growth for next 5 days. The root length was recorded on the 6^th^ day post transfer.

### Measurements of transpirational WLR and RWC

The water loss rate of detached leaf is a critical method as described by [88] to determine transgenic plants water status under imposed stress condition. The water loss rate (WLR) was determined by using detached leave from 10 plants of *GaMYB62L* lines and WT plants. Four week test and control plants leaves were detached and fresh weight (FW) were scored, afterwards left on bench at 22°C RT with humidity level of 45–50%, then leave weights were recorded at designated time. The relative water content of leaves was calculated by [74] method. The cut leave were then submerged in water for next 4 h, to get turgid weights. At last, after drying leave for 72h at 80°C in an oven, dry weight of leave were recorded. RWC percentage = (Fresh weight (FW) − Dry weight (DW))/ (Turgid weight (TW) − Dry weight (DW)) ×100 formula used to calculate relative water content for test and control samples.

### Analysis of proline content and chlorophyll content

Endogenous plant proline content was measured using PRO Kit (Jiancheng Bioengineering Institute, Nanjing, China). 0.2 g of Leaf segments were homogenized with 1.8ml Solution I followed by centrifugation at 3500g for 10 min. 250μl of sample supernatant, 0.5 μl of both Sol. I and Sol. II were mixed together and boiled for 30 min, and finally the absorbances were determined at 520nm.

The determination of chlorophyll content was done by using 0.1 g of rosette leaves in 1.5 ml 95% ethanol at RT. The chlorophyll content extraction was carried out in dark place. The absorbances of chlorophyll extracted from test and control plants were checked at wavelength 649 and 665 nm. Total chlorophyll *(a+b)* concentration was monitored as follow: ((OD665nm×13.95−OD649nm×6.88) + (OD 649nm×24.96−OD665nm×7.32))/ (sample weight) [89]. Test and control samples were measured thrice and the results were averaged.

### Stomata opening rate determination in response to Abscisic acid treatment

Three week old plants leaves were dipped in the stomatal opening solution: 10mMol/L CaCl_2_, 50mMol/L KCl and 5 mMol/L MES with, pH 6.15 [90] for 2h in a lighted growth chamber with 95±5% RH. Stomata apertures measurements were done after 2.5h; with application of two different concentrations of Abscisic acid (ABA) (5 and 10 μmol/L). Control experiments were performed without ABA.

### Gene expression analysis of markers genes by qRT-PCR

Marker genes analysis was done by using 14 d seedlings of test and control plants treated with 100μM ABA. RNA extractions were done by RNA-prep Pure Plant kit (Tiangen, China), while cDNA were synthesized by PrimeScript RT Master Mix (Takara, Clontech, China). The SYBR Premix ExTaq^™^ (Takara, Clontech, China), and 7900HT detection system by (Applied Biosystem) were used for qRT-PCR analysis.

The qRT-PCR programme was as follow 95°C for 30 sec, 95°C for 5 sec (40 cycles), 60°C for 30 sec, and 95°C for 15 sec, followed by 60°C for 15 sec. The Arabidopsis *Ubiquitin 10 gene* was used as control gene with (*Accession no*: *AT4G05320*). The relative transcripts detected for gene was computed by using the relative 2^-ΔΔCt^ method [91], with error bars which indicate SD value (*n* = 3). Three biological replicates were performed using independent cDNA preparations.

### Bioinformatic analysis of *GaMYB62L* gene

The conserved domain structure analysis was performed by (http://www.ncbi.nlm.nih.gov). Motif analysis of gene sequence was done by SMART (http://asp.pku.edu.cn) and GENE Structure Display Server was used intron exon analysis (http://gsds.cbi.pku.edu.cn). *GaMYB62L* polypeptide physicochemical properties like MW and pI were analyzed by ExPASy (http://www.expasy.org/proteomics), online proteomics tool. Transmembrane domains prediction was produced using the TMHMM 2.0 portal (http://www.cbs.dtu.dk). Also, *GaMYB62L* protein secondary structure was calculated through protein homology & analogy portal (https://npsa-prabi.ibcp.fr/cgi-bin/secpred_gor4.pl/) recognition engine (S1A–S1F Fig). The multiple sequence alignment of *GaMYB62L* R2R3 homolog was performed by EMBL-EBI Muscle online tool (http://www.ebi.ac.uk/Tools/msa/muscle/). The phylogenetic tree analysis of retrieved homologous proteins was carried out by using NJ-method in MEGA 6.0 program [92]. The Bootstrap values with 1000 replicate were used to evaluate the strength of nodes in the branch of tree.

### Statistical analysis of Data

All investigational data were the means of three independent experiment replicates, and results were determined using analysis of variance and student t-test. Variation among treatment means of control and test of proline and chlorophyll content were evaluated using Duncan’s multiple range tests.

## Supporting Information

S1 Table Predicted *Cis*-acting elements in *GaMYB62L* promoter region. (DOCX)

S2 Table Survival (%) of *GaMYB62L* transgenic plants in 6% BASTA selection medium. (DOCX)

S3 Table The primers sequences used for *GaMYB62L*. (DOCX)

S1 Fig Bioinformatic analysis of GaMYB62L. (DOCX)

S2 Fig Survival rate in BASTA selection of transformants and PCR confirmation of *GaMYB62L* over- expression in transgenic plants. (DOCX)
