# Supplementary material for: Functional Characterization of Cotton GaMYB62L, a Novel R2R3 TF in Transgenic Arabidopsis
Source: PLoS One. 2017 Jan 26;12(1):e0170578. doi: 10.1371/journal.pone.0170578 (PMC5268478; doi:10.1371/journal.pone.0170578)
Supplement: S1 Table — (DOCX) [file pone.0170578.s001.docx]

**Supplementary Tables**

**S1 Table. Predicted *Cis*-acting elements in *GaMYB62L* promoter region**

|  | **Element** | **Sequence** | **Function** |
| --- | --- | --- | --- |
| **Stress** | HSE | AAAAAATTTC | cis-acting element involved in heat stress responsiveness |
|  | Box-W1 | TTGACC | fungal elicitor responsive element |
|  | HSE | AAAAAATTTC | cis-acting element involved in heat stress responsiveness |
|  | MBS | CAACTG | MYB binding site involved in drought-inducibility |
|  | TC-rich repeats | ATTTTCTTCA | cis-acting element involved in defense and stress responsiveness |
|  | WUN-motif` | AAATTTCCT | wound-responsive element |
| **Development** | AT1-motif | ATTAATTTTACA | part of a light responsive module |
|  | Box 4 | ATTAAT | part of a conserved DNA module involved in light responsiveness |
| ` | Box 4 | ATTAAT | part of a conserved DNA module involved in light responsiveness |
|  | Box I | TTTCAAA | light responsive element |
|  | AT1-motif | ATTAATTTTACA | part of a light responsive module |
|  | CAT-box | GCCACT | cis-acting regulatory element related to meristem expression |
|  | G-Box | CACGTT | cis-acting regulatory element involved in light responsiveness |
|  | G-Box | CACGTT | cis-acting regulatory element involved in light responsiveness |
|  | G-box | CACATGG | cis-acting regulatory element involved in light responsiveness |
|  | G-box | GACATGTGGT | cis-acting regulatory element involved in light responsiveness |
|  | 3-AF1 binding site | TAAGAGAGGAA | light responsive element |
|  | chs-CMA2a | TCACTTGA | part of a light responsive element |
|  | circadian | CAANNNNATC | cis-acting regulatory element involved in circadian control |
|  | circadian | CAANNNNATC | cis-acting regulatory element involved in circadian control |
|  | chs-CMA2a | TCACTTGA | part of a light responsive element |
|  | circadian | CAANNNNATC | cis-acting regulatory element involved in circadian control |
| **Hormone** | CGTCA-motif | CGTCA | cis-acting regulatory element involved in the MeJA-responsiveness |
|  | CGTCA-motif | CGTCA | cis-acting regulatory element involved in the MeJA-responsiveness |
|  | CGTCA-motif | CGTCA | cis-acting regulatory element involved in the MeJA-responsiveness |
|  | TGACG-motif | TGACG | cis-acting regulatory element involved in the MeJA-responsiveness |
|  | TGACG-motif | TGACG | cis-acting regulatory element involved in the MeJA-responsiveness |
|  | TATA-box | TATA | core promoter element around -30 of transcription start |
|  | CAAT-box | CAAT | common cis-acting element in promoter and enhancer regions |
|  | CAAT-box | CAAT | common cis-acting element in promoter and enhancer regions |

*GaMYB62L* promoter *cis-*element prediction by Plant CARE (http://bioinformatics.psb.ugent.be/webtools/).
