# Supplementary material for: Functional Characterization of Cotton GaMYB62L, a Novel R2R3 TF in Transgenic Arabidopsis
Source: PLoS One. 2017 Jan 26;12(1):e0170578. doi: 10.1371/journal.pone.0170578 (PMC5268478; doi:10.1371/journal.pone.0170578)
Supplement: S2 Table — (DOCX) [file pone.0170578.s002.docx]

**S2 Table. Survival (%) of *GaMYB62L* transgenic plants in 6% BASTA selection medium.**

| **Representative Lines** | **T2 generation**  **Survival (%)** | **T2 generation**  **Dead (%)** | **T3 generation**  **Survival (%)** |
| --- | --- | --- | --- |
| 1 L1 | 77 | 23 | 100 |
| 2 L2 | 68 | 32 | 100 |
| 3 L3 | 76 | 24 | 100 |

**(A)** Survival (%) of *GaMYB62L* transgenic plants in MS with 6% BASTA. At T_2_ generation of transgene, the segregated ratio of survived to dead plant was (3:1) in 6% BASTA medium plates. The survival rate of T_3_ seeds was 100% in 6% BASTA medium plates. The values are from mean germination rate (%), 100 seeds per plate.
