# Supplementary material for: Functional Characterization of Cotton GaMYB62L, a Novel R2R3 TF in Transgenic Arabidopsis
Source: PLoS One. 2017 Jan 26;12(1):e0170578. doi: 10.1371/journal.pone.0170578 (PMC5268478; doi:10.1371/journal.pone.0170578)
Supplement: S3 Table — (DOCX) [file pone.0170578.s003.docx]

**S3 Table. The primers sequences used for *GaMYB62L***

| **Primer name** | **Primer Sequence 5’to3’** | **Function** |
| --- | --- | --- |
| GaMYB62L | F: TCTAGAATGAGATTAATCGGTGAGAT  R: GAGCTCTCAATGCAATGCATTTCTAA | For ORF amplification |
| 35S primer (F)  GaMYB62L(R) | F: GACGCACAATCCCACTATCC  R: GAGCTCTCAATGCAATGCATTTCTAA | Test of transgenic Arabidopsis T_0_ |
| qGaMYB62L | F: GCCTGGAAGAACCGATAATGAGA  R:ATCGGAGAATGTTCTTAAACGCC | qRT-PCR of T_2_ transformant lines |
| Ubiquitin 10 | F: AACTTTGGTTTGTGTTTTGG  R:TCGACTTGTCATTAGAAAGAAAGAGATAA | qRT-PCR control gene |
| ABI1 | F: AGCTGCTGATATAGTCGTCGTTGATA  R: GAGGATCAAACCGACCATCTAACA | Marker gene for qRT-PCR |
| ABI2 | F: GTTCTTGTTCTGGCGACGGAGC  R: CCATTAGTGACTCGACCATCAAG | Marker gene for qRT-PCR |
| RD22 | F: ACTTGGTAAATATCACGTCAGGGCT  R: CTGAGGTGTTCTTGTGGCATACC | Marker gene for qRT-PCR |
| RD29A | \| F:GATAACGTTGGAGGAAGAGTCGG \| \| --- \|   R:TCCTGATTCACCTGGAAATTTCG | Marker gene for qRT-PCR |
| RD29B | F: CCGACAAGAGGTGATGTGAAAGTAG  R:GTGTAACCTAGCTTTGAGGCAACG | Marker gene for qRT-PCR |
| P5CS | F: GAGCAATGGAGTCACTTTGTATGG  R: TTCCTCTCATTATCCATCTCGTTG | Marker gene for qRT-PCR |
| ADH | F: ATGAAGCTGGAGGGATTGTTGAG  R: AGAGGAGCATCCGGATTGATCTTA | Marker gene for qRT-PCR |
| COR15A | F: GTGACGGATAAAACAAAAGAGG  R: GACCCTACTTTGTGGCATCCTT | Marker gene for qRT-PCR |
| EM6 | F: CTCAACAAGAGAAGAAGCAGCTGG  R: GGTCTTGGTCCTGAATTTGGATT | Marker gene for qRT-PCR |
| RD26 | F: GACCCAATAGAGTAGCCGGG  R: TTTTTAATCCCGACACGACG | Marker gene for qRT-PCR |
