# Supplementary material for: Functional Characterization of Cotton GaMYB62L, a Novel R2R3 TF in Transgenic Arabidopsis
Source: PLoS One. 2017 Jan 26;12(1):e0170578. doi: 10.1371/journal.pone.0170578 (PMC5268478; doi:10.1371/journal.pone.0170578)

**S1Fig.** Bioinformatic analysis of *GaMYB62L* **(A)** Conserved domains of *GaMYB62* from *G.arboreum* **(B)** Predicted protein structure of *GaMYB62L* by SMART. Two *SANT* domain (1) 51 AA and (2) 49 *AA, SWI3, ADA2, NCoR* and *TFIIIB DBD* with (E value: 8.6e_14) and (1.45e_15) respectively; 2 low complexity segments with 12 and 19 AA respectively **(C)** Intron Exon analysis by online GSD Server **(D)** *GaMYB62L* TMH prediction by TMHMM 2.0 version online Server. Intracellular loop and extracellular loops were shown by a blue and red colored line **(E)** Computation of pI/MW of *GaMYB62L* by proteomics ExPASy website. **(F)** Secondary Structure calculation by GOR ExPASy online website.


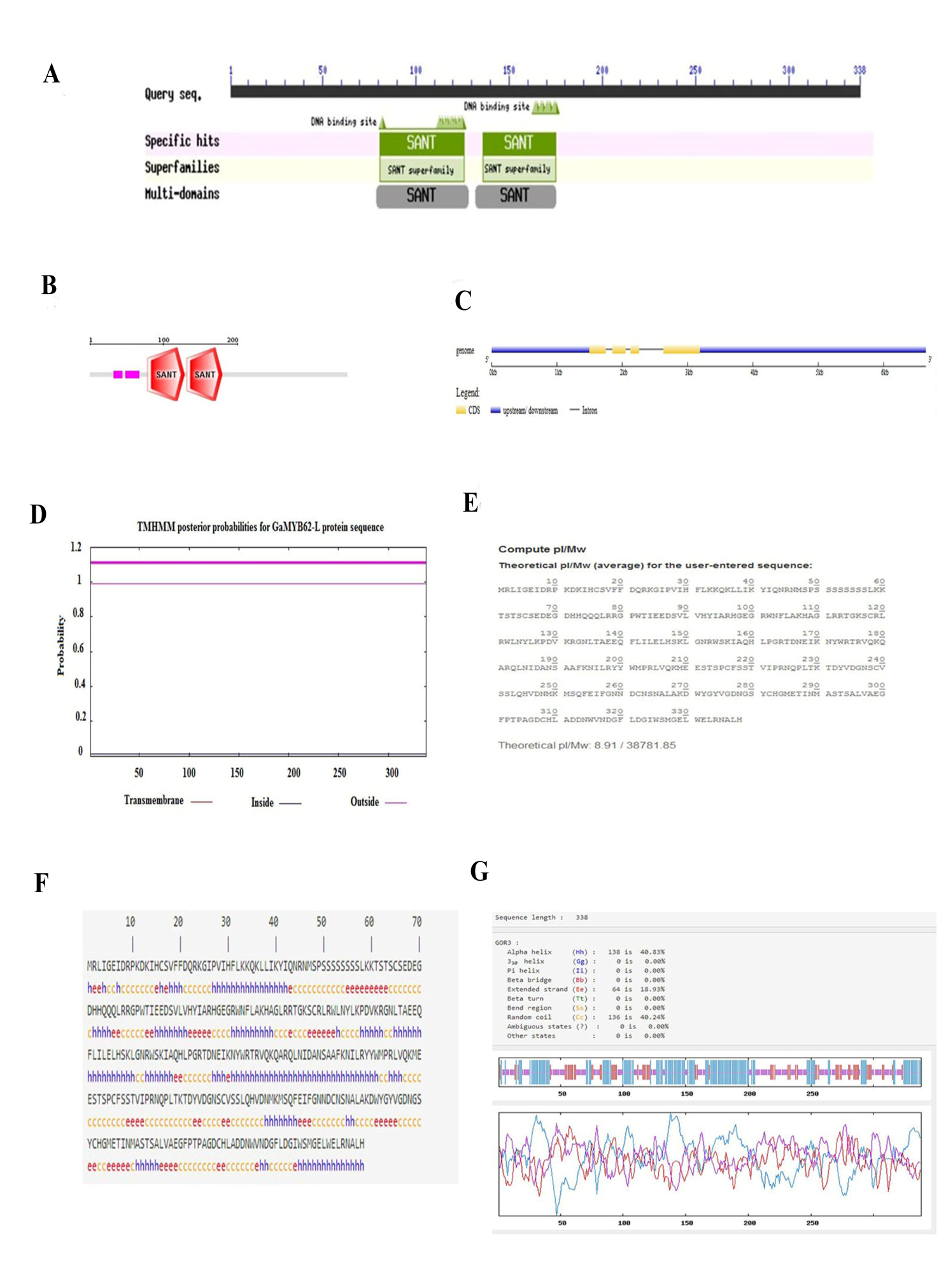

Supplement: S1 Fig — (DOCX) [file pone.0170578.s004.docx]
