# Supplementary material for: Functional Characterization of Cotton GaMYB62L, a Novel R2R3 TF in Transgenic Arabidopsis
Source: PLoS One. 2017 Jan 26;12(1):e0170578. doi: 10.1371/journal.pone.0170578 (PMC5268478; doi:10.1371/journal.pone.0170578)

**S2 Fig. Survival rate in BASTA selection of transformants and PCR confirmation of *GaMYB62L* over- expression in transgenic plants**

**(A)** And **(B)** *GaMYB62L* T_0_ transgenic plants survival rate after 1% BASTA spray. Seeds sown in the soil pots, then after one week seedlings growth, sprayed with 1% BASTA solution twice, with 3 days interval and covered with polythene sheet for 6 hr and then grew normally. Negative plants become pale and dead while transformed one stayed green and grew normal. Positive seedlings shifted to new pots and set to get T_1_ seeds. Representative plants were photographed after 18 d germination in soil after 1% BASTA spray. WT were grown without BASTA spray as control. **(C)** And **(D)** The survival rate of WT and *GaMYB62L* T_3_ transgenic seeds on selective medium containing BASTA. Representative plant was photographed after 8 days of germination in 6% BASTA media plates. **(E)** *GaMYB62L* gene confirmation in transgenic Arabidopsis plants. Genomic DNA from T_1_ generation leaves was extracted by using Mighty Amp Genotyping Kit, *35S* as forward primer and reverse gene specific primer was used for template amplification. Lane 1: DNA Marker III; lane 22: WT as negative control.


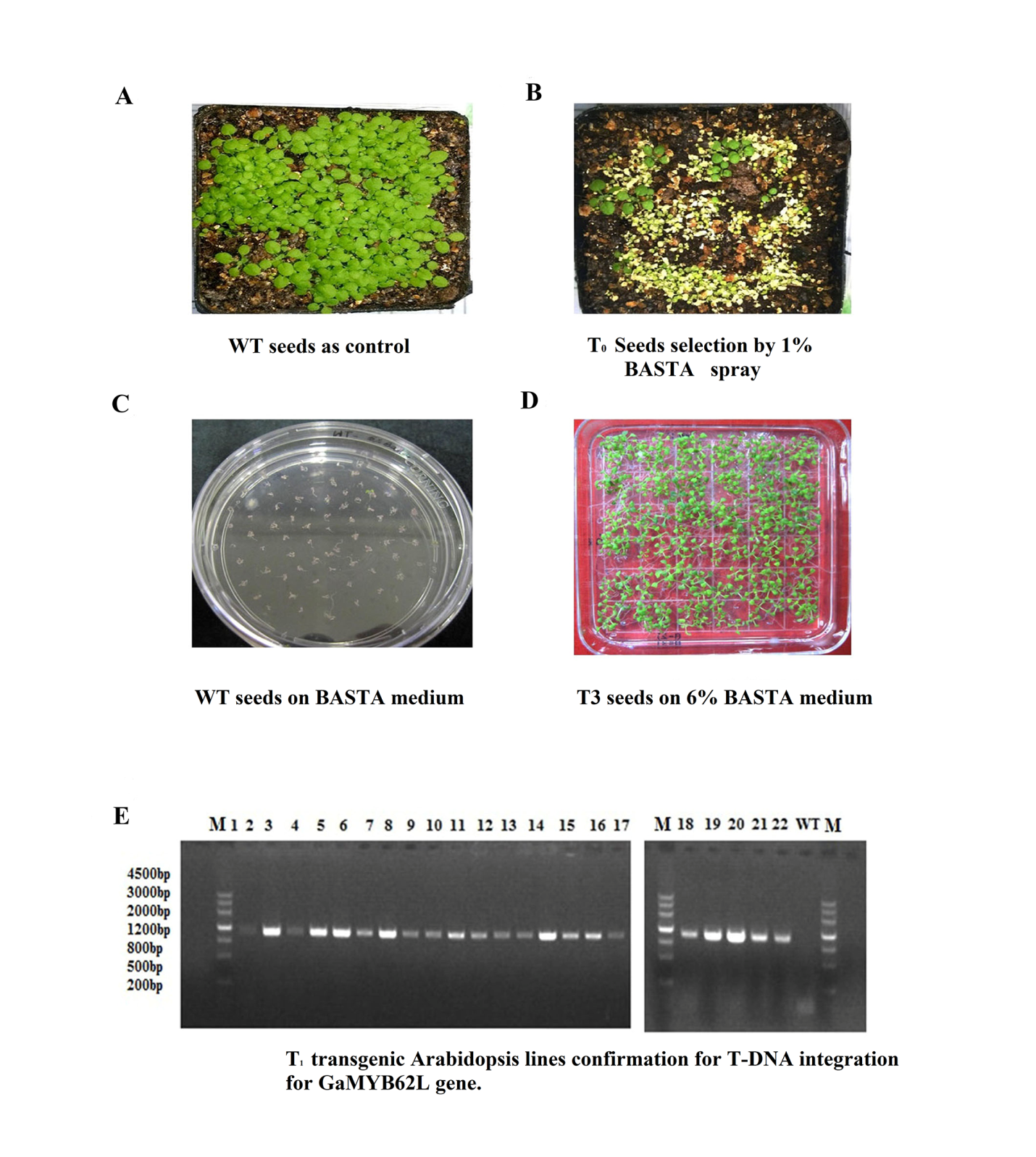

Supplement: S2 Fig — (DOCX) [file pone.0170578.s005.docx]
